# Participant perceptions on the acceptability and feasibility of a telemedicine-based HIV PrEP and buprenorphine/naloxone program embedded within syringe services programs: a qualitative descriptive evaluation

**DOI:** 10.1186/s12954-022-00718-1

**Published:** 2022-12-03

**Authors:** Amy Corneli, Brian Perry, Andrea Des Marais, Yujung Choi, Hillary Chen, Rebecca Lilly, Denae Ayers, Jesse Bennett, Lauren Kestner, Christina S. Meade, Nidhi Sachdeva, Mehri S. McKellar

**Affiliations:** 1 Department of Population Health Sciences, Duke University School of Medicine, 215 Morris St, Durham, NC 27701 USA; 2 Duke Clinical Research Institute, Duke University School of Medicine, Durham, NC USA; 3 Department of Medicine, Duke University School of Medicine, Durham, NC USA; 4 North Carolina Harm Reduction Coalition, Wilmington, NC USA; 5 Queen City Harm Reduction, Charlotte, NC USA; 6 Department of Psychiatry and Behavioral Sciences, Duke University School of Medicine, Durham, NC USA; 7 Present Address: Port City Harm Reduction, Wilmington, NC USA; 8 Present Address: North Carolina Association of County Commissioners, Raleigh, NC USA

**Keywords:** Pre-exposure prophylaxis, HIV prevention, People who inject drugs, Medication for opioid use disorder, Telemedicine, Syringe services programs

## Abstract

**Background:**

People who inject drugs (PWID) are at risk for HIV and opioid overdose. We piloted PARTNER UP, a telemedicine-based program to provide PWID with access to both oral pre-exposure prophylaxis (PrEP) for HIV prevention and medication for opioid use disorder (MOUD) through two syringe services programs (SSPs) in North Carolina. We conducted a qualitative evaluation to assess the acceptability and feasibility of PARTNER UP from the participant perspective.

**Methods:**

PARTNER UP participants met with a provider for an initial in-person visit at the SSP, followed by weekly telemedicine visits in month 1 and then monthly telemedicine visits until program end at month 6. Using a qualitative descriptive study design, we conducted in-depth interviews with a subsample of PARTNER UP participants at 1 month and 4 months. Informed by the technology acceptance model, we assessed participant perceptions of the usefulness and ease of use of PARTNER UP, as well as their intent to continue to use the program’s components. We audio-recorded all interviews with participants’ permission and used applied thematic analysis to analyze the verbatim transcripts.

**Results:**

We interviewed 11 of 17 people who participated in PARTNER UP—10 in the month 1 interview and 8 in the month 4 interview. Nearly all participants were motivated to join for consistent and easy access to buprenorphine/naloxone (i.e., MOUD); only a few joined to access PrEP. Most were comfortable accessing healthcare at the SSP because of their relationship with and trust toward SSP staff, and accessing services at the SSP was preferred compared with other healthcare centers. Some participants described that telemedicine allowed them to be honest and share more information because the visits were not in-person and they chose the location, although the initial in-person meeting was helpful to build provider trust and rapport. Most participants found the visit schedule to be feasible, although half described needing to reschedule at least once. Nearly all participants who were interviewed intended to continue with MOUD after the program ended, whereas none were interested in continuing with PrEP.

**Conclusions:**

Participant narratives suggest that the PARTNER UP telemedicine program was acceptable and feasible. Future studies should continue to explore the benefits of embedding both PrEP and MOUD into SSPs with larger numbers of participants.

*Trial registration* Clinicaltrials.gov Identifier: NCT04521920.

## Background

People who inject drugs (PWID) are at risk for HIV if they share syringes and other injection equipment and/or have sex with individual(s) with untreated HIV [1]. Several approaches are available to reduce HIV risk among PWID, including oral pre-exposure prophylaxis (PrEP), a daily pill. PrEP with tenofovir disoproxil fumarate (TDF) has been shown to be safe and effective among PWID [2]. Despite approval by the US Food and Drug Administration in 2012, awareness [3–5] and use [4–6] of PrEP among PWID is currently limited, although PWID have reported interest in or willingness to use PrEP [5–8] or viewed it positively after learning about it [3]. Stigma [8–10], negative provider interactions [9], limited provider willingness to provide PrEP to PWID [11], difficulties in accessing healthcare [7], and costs/co-pays [6, 7] have been identified as factors that may make it difficult for PWID to obtain PrEP care.

PWID have suggested or supported combining PrEP services with other PWID-related services, such as syringe services programs (SSPs) [7, 8, 10]. SSPs are programs that reduce PWID’s risk of acquiring HIV and other blood-borne infections, such as hepatitis C, by providing unused syringes and injection equipment, education, and referrals for testing and treatment [12]. Compared with traditional medical centers, SSPs are often community based, staffed by peers of PWID, and serve as a trusted source of non-judgmental and non-stigmatizing support and resources for PWID [8, 10, 13].

The role of SSPs in ending the HIV epidemic in the USA will be most impactful—and can best support PWID’s overall health and well-being [14]—when they expand their risk and harm reduction approaches to include PrEP and MOUD [15]. Because PWID are also at risk for overdose if they inject opioids [16], combination programs can address both HIV prevention and opioid use disorder. Medication for opioid use disorder (MOUD) can reduce all-cause mortality among PWID [17], and use of MOUD has been shown to reduce HIV risk [18–21]. Yet, as with PrEP, uptake of MOUD in PWID is also limited. More than a quarter of PWID responding to a recent national survey reported trying but being unable to access MOUD [22]. Factors that contribute to limited MOUD use are similar to PrEP and include stigma about MOUD [23, 24], high out-of-pocket costs—particularly for uninsured patients [23, 25, 26], limited awareness of treatment locations [26], lack of providers [25, 27, 28], and prior negative healthcare experiences [23, 26].

Embedding PrEP and MOUD programs in SSPs is a promising comprehensive HIV prevention approach to address shared barriers and improve PWID’s knowledge of, access to, and uptake of both PrEP and MOUD. To date, a small number of SSPs have provided PrEP or MOUD through collaboration or integration with medical providers [29–35]. Nearly all of these programs, however, have focused on providing access to either PrEP or MOUD and have not offered access to both medications in a single program or offered services via telemedicine.

We created PARTNER UP (Providing A Resource: Telemedicine at Needle Exchanges for Underserved Populations) to address this gap. PARTNER UP provided PWID with access to both oral PrEP and MOUD and telemedicine visits with a provider through two SSPs in North Carolina. Since the North Carolina legislature legalized SSPs in 2016, nearly 50 SSPs have been registered across the state [36]. In 2020, we initiated PARTNER UP in two well-established SSPs—one in Charlotte, the largest metropolitan area in North Carolina, and the other in Wilmington, a smaller coastal city. Charlotte is located in Mecklenburg County, a priority jurisdiction for the US Government’s Ending the HIV Epidemic Initiative given the high rate of new HIV diagnoses in the USA [37]. Wilmington is located in New Hanover County, which has a high rate of opioid overdose deaths [38]. We added a telemedicine component to increase feasibility and scalability for providing services in multiple locations across the state. Telemedicine allows providers to be “present” in a non-clinical site, such as an SSP or the patient’s home, and has been found to be comparable to in-person treatment for opioid use disorder [39–42]. Additionally, providing telemedicine services using a computer at an SSP or on a patient’s own device may improve appointment attendance by providing more convenience to the patient [39, 43, 44].

The purpose of the PARTNER UP pilot was to assess: (1) PrEP and MOUD uptake and retention in treatment, and (2) program acceptability and feasibility among participants. A brief description of the pilot and findings on PrEP and MOUD uptake and retention has been previously described [45, 46], and a detailed description of program outcomes will be published elsewhere. In this article, we focus on participant perceptions on the acceptability and feasibility of PARTNER UP.

## Methods

### Description of the pilot program and participants

In addition to following standard medical guidelines used in clinical practice, individuals were eligible to participate in the pilot if they were 18 years of age and older, had a confirmed HIV-negative status, were not currently taking PrEP, were not consistently taking MOUD, were at risk for HIV due to a history of sharing injection or drug preparation equipment or engaging in sexual behaviors associated with increased HIV risk, and had self-reported a history of opioid injection in the past 6 months. To enroll, participants must have been willing to take both PrEP (emtricitabine/tenofovir disoproxil fumarate [FTC/TDF], brand name Truvada^®^) and MOUD (buprenorphine and naloxone [bup/nx], brand name Suboxone^®^); however, participants were informed that they could stop taking either medication at any time and remain in the study. Individuals who were uninterested in PrEP and interested in MOUD at enrollment were linked to other MOUD programs in their region, mostly methadone programs. We designed the PARTNER UP pilot to be small, with up to 20 participants, to gather proof-of-concept findings on integrating PrEP and MOUD services in SSPs. A total of 17 PWID participated in PARTNER UP. At enrollment in the PARTNER UP program, 94% (*n* = 16) reported having used an opioid in the past month and 94% (*n* = 16) also reported ever taking buprenorphine prior to enrolling in PARTNER UP [45, 46].

PARTNER UP was conducted during the COVID-19 pandemic, and the SSPs remained open during this time. The initial visit with the study’s healthcare provider was in-person at the SSP, as we believed it was critical to establish rapport between the participant and the provider at the beginning of the program. Prescriptions for PrEP and MOUD were sent to a partnering retail pharmacy in the area for in-person pickup. Participants could also choose to have PrEP directly mailed from a pharmacy to their homes. When participants did not have insurance, the cost for MOUD was covered by the study and PrEP was covered by the manufacturer’s patient assistance program. The study also covered medication co-pays for insured participants when necessary. All follow-up visits were conducted using telemedicine. Participants could choose whether to have these visits via video conferencing on a computer at the SSP—or on the participant’s smartphone, tablet, computer, or telephone at the location of their choice. Participants could also utilize the MyChart patient portal, which was installed on their phones, for messaging with the provider (through the Epic electronic health record), and automated appointment reminders were sent via phone call, text, or email. Participants were asked to attend weekly visits for the first month while they were stabilized on their MOUD dose, followed by monthly visits for a total of 6 months. Laboratory assessments were conducted at baseline, 3 months, and 6 months and included blood testing for HIV, hepatitis B and C, and a comprehensive metabolic panel, and urine pregnancy testing for individuals with a uterus. At completion of the pilot, participants who were interested in continuing PrEP and/or MOUD were referred to local providers for continued access at the completion of the pilot.

### Evaluation study design and data collection

Using a qualitative descriptive study design [47, 48], we conducted in-depth interviews (IDIs) with a subsample of PARTNER UP participants. We aimed to interview 10 participants at month 1 of program participation and 10 participants at month 4, with 5 participants per site per time point. Our sampling strategy included both random sampling (month 1) and purposive sampling [49] (month 4) of participants who had not been interviewed at month 1 in order to gather as many unique perspectives as possible.

Our inquiry was informed by the original version of the technology acceptance model (TAM) [50]. TAM examines how individuals adopt technology, and the model has been used in research, including descriptive research, on the use of patient portals, mobile apps, and telemedicine, for example [51–55]. We used the TAM domains for both the technology and medication components of the program. To explore participants’ perceptions on the acceptability of the PARTNER UP program, IDI questions focused on the TAM domain “perceived usefulness,” which we defined as motivation to join the program, the utility of combining access to PrEP and MOUD in a single program, the appropriateness of embedding PARTNER UP at an SSP and in using telemedicine, and the benefits gained from participation. To explore participants’ perceptions on the feasibility of program participation, the IDI questions focused on the TAM domain “perceived ease of use,” which we defined as perceptions on the frequency of the telemedicine visits and the ease or difficulty of accessing PrEP and MOUD. We also directly explored the TAM domain “intent to use,” which we defined as reasons to continue/not continue with the program (at the month 1 interview) and expected PrEP and MOUD use after program completion (at the month 4 interview). In an effort to reduce socially desirable responses, the IDIs were conducted by a trained qualitative interviewer not associated with the implementation of the pilot program; participants were also informed that the program’s healthcare provider did not have access to their individual interview responses. All IDIs were conducted by telephone and audio-recorded with participant permission from December 2020 to June 2021.

### Data analysis

We used principles of applied thematic analysis [56] to analyze the data and NVivo 12 [57] to apply codes to verbatim transcripts. After the month 1 data collection was complete, three analysts (including the interviewer) reviewed a sample of the transcripts and developed an a priori topic-based codebook derived from the interview guide. The analysts piloted the codebook by independently coding two transcripts (one from each site) and met to discuss their application of the codes. Any discrepancies were discussed, and revisions to the codebook were made. One analyst then applied the topic-based codebook to the remainder of the transcripts. The three analysts then reviewed the data related to each topic and independently identified salient information informing the topic. Analysts met to discuss the identified information and to develop a content-based codebook. The drafted codebook was piloted on 20% of the data related to the topic by the three analysts using the same process as the topic-based codebook. After the content-based codebook was finalized, one analyst continued to code the remainder of the text. The same process for codebook development and coding was used for the month 4 IDIs. After all coding was complete, coding frequency tables were generated to identify the most salient content-based themes that emerged across the dataset at each time point (month 1 IDIs and month 4 IDIs). Summaries of the emergent themes for each topic were created, including illustrative quotes from participants for key themes. Of note, participants referred to the MOUD as “Suboxone” in the IDIs and hence we use that term when describing participant narratives.

### Ethics

The Duke University Health Sciences Institutional Review Board approved the study. We created a separate protocol for the acceptability and feasibility study to reduce social desirability bias. Participants provided their oral informed consent for participating in the IDIs and were compensated with a $20 gift card for each interview.

## Results

### Study population

Of the 17 PWID who participated in the PARTNER UP program, 11 participated in the IDIs: 10 in the IDI at 1 month and 8 in the IDI at 4 months. Of these individuals, 7 participated in both IDIs. Interview participants were ages 24 to 71, and most were non-Hispanic White men (*n* = 8, 73%). Almost half had completed some college (*n* = 5, 45%) and around a quarter (*n* = 3, 27%) completed some but did not graduate from high school. At the time of enrollment, over half were single (*n* = 6, 55%) and just over a third (36%) were in a relationship or married. Additionally, just over a third of participants (*n* = 4, 36%) were unemployed or unable to work, and almost half (*n* = 5, 45%) were employed either part- or full-time (Table 1).

**Table 1:** Participant descriptive information, *n* (%)

Characteristic*, **	Site		Total *n* = 11
	Wilmington *n* = 5	Charlotte *n* = 6	
*Age, in years*			
20–29	1 (20.0)	1 (16.7)	2 (18.2)
30–39	3 (60.0)	2 (33.3)	5 (45.5)
40–49	0 (0.0)	2 (33.3)	2 (18.2)
50 +	1 (20.0)	1 (16.7)	2 (18.2)
*Gender identity*^*a*^			
Male	4 (80.0)	5 (83.3)	9 (81.9)
Female	1 (20.0)	1 (16.7)	2 (18.2)
*Race*			
White	5 (100.0)	6 (100)	11 (100)
*Ethnicity*			
Not Hispanic or Latino	5 (100.0)	5 (83.3)	10 (90.9)
Hispanic or Latino	0 (0.0)	1 (16.7)	1 (9.1)
*Highest grade or year of school completed*			
Some high school	1 (20.0)	2 (33.3)	3 (27.3)
High school or GED	1 (20.0)	1 (16.7)	2 (18.1)
Some college	3 (60.0)	2 (33.3)	5 (45.5)
Associate's degree	0 (0.0)	1 (16.7)	1 (9.1)
*Marital/partnership status*			
Single and never married	3 (60.0)	3 (50.0)	6 (54.5)
Legally married/legal domestic partnership	0 (0.0)	2 (33.3)	2 (18.2)
Partnered and living together, or informally married	1 (20.0)	1 (16.7)	2 (18.2)
Separated	1 (20.0)	0 (0.0)	1 (9.1)
*Work status*			
Unemployed	1 (20.0)	2 (33.3)	3 (27.3)
Working part-time^b^	2 (40.0)	1 (16.7)	3 (27.3)
Working full-time^c^	1 (20.0)	1 (16.7)	2 (18.2)
Disabled, not able to work	0 (0.0)	1 (16.7)	1 (9.1)
A full-time, stay-at-home parent	0 (0.0)	1 (16.7)	1 (9.1)
Full-time student	1 (20.0)	0 (0)	1 (9.1)

*Demographic information collected as part of main study

**Some totals do not equal 100% due to rounding

^a^Gender options: male, female, transgender male, transgender female, non-binary/gender non-conforming, other

^b^Less than 35 h a week, includes labor pool, day work, and self-employed

^c^35 hours or more a week, includes self-employed

### Acceptability—perceived usefulness of PARTNER UP

Nearly all participants said they were motivated to join PARTNER UP to gain consistent and easy access to Suboxone^®^. Several described their history of accessing Suboxone^®^ prior to enrolling in PARTNER UP, which was primarily on the street or at a “detox center,” and shared that access was often unreliable or expensive. A 50-year-old male explained:

I decided to take part in the program because [in my previous program], you had to go every day to get your Suboxone^®^ dose…You can’t get a prescription…And it’s really a struggle to get there every day. You gotta go. You gotta wait in line...There’s a bunch of people that don’t wanna be part of the program, so you’re just really surrounded by not good things. So, when I found out through a friend of mine that Harm Reduction [i.e., the SSP] was doing this program where you could go in and do a telemed thing, and they would monitor your prescription and prescribe to you long-term, like a month at a time, the Suboxone^®^ at a regular dose, I was like, “That’s a godsend. That’s exactly what I’ve been looking for.” So, it just really makes the access to the medicine that I think I’m gonna need long-term very easy for me.

Another participant, a 30-year-old male, emphasized that being able to access Suboxone^®^ legally through PARTNER UP was a motivating factor:

I’m mostly [participating] for the Suboxone^®^. I had been taking it before, and I had a prescription for it. I lost my health insurance and wasn’t able to take it legally anymore, so I had to get it off the street. When I heard about the program, I was like, “Oh, cool. A good legal way to get it and get it consistently.”

Two participants said they joined PARTNER UP to access PrEP, with one specifying they joined only for PrEP. At the beginning of the program, some participants said they understood that their past drug use and sexual behaviors put them at risk for HIV, and if those behaviors continued, they would be interested in taking PrEP. However, more participants described not perceiving they were at risk for HIV at the time of the interview, and most viewed taking PrEP as a requirement of program participation in which they were willing to oblige to get access to Suboxone^®^. Two participants elaborated:

I guess it would be more to help out with the study [i.e., why I’m taking PrEP]…It was a packaged deal honestly, to help out, I just kinda took it (i.e., PrEP) to get in with the Suboxone^®^, to be honest… I probably wouldn’t have (otherwise) ‘cause I have no run-ins with HIV, no one in my family, no one else I know. —a 35-year-old male

There’s no way that I would ever consider taking PrEP if I wasn’t in the study…I feel like that being able to be part of the program has changed my life and given me an opportunity that I didn’t have. And that’s kind of why I said I would take the Truvada^®^ [i.e., PrEP] anyway even though I don’t feel like I need it is because I know that that’s part of the study, and it’s required in order to get what I need. So, I feel like taking the Truvada^®^ [i.e., PrEP] is the least I can do for them providing me with an opportunity to change my life. You know what I mean? —a 34-year-old female

Participants described the usefulness of offering PrEP at the SSP even though they were not personally interested. Some elaborated that it is likely helpful in making PWID aware of PrEP because they may know others in their social network who could benefit from learning about PrEP. A 25-year-old female said:

I can see how that [offering PrEP] would be beneficial for a lot of people. Especially if this program eventually can reach the participants that could actually benefit from that aspect of it a little bit more. So, sex workers and stuff like that. Or anybody, I mean IV drug users absolutely too. People who are just maybe sharing more supplies and stuff are definitely at risk for that. But personally, I’m not. But that doesn’t mean that someone else in the program—or when this gets expanded, which hopefully it will—would definitely find that beneficial.

Most participants explained that embedding PARTNER UP within an SSP made them more comfortable initiating and accessing healthcare services compared with programs offered at other healthcare facilities, primarily because of their prior relationship with SSP staff. Some participants elaborated that because of this relationship, SSP staff were aware of their interest in MOUD and therefore able to refer them to the PARTNER UP program.

I think it would be fair to say that…it can be really uncomfortable to go somewhere that you’re not familiar [with], especially as a drug user. Or as a sex worker. So, I think that because it’s a place that people can be familiar with, that they’re gonna feel less judged or just a little more comfortable in general. Because it’s a location that they may be familiar with. —a 25-year-old female

Well, I like the fact that we are already familiar with the people at [the SSP]. So, to kind of move into another area of treatment through them was more comfortable for us. For me, especially, I like that we were able to go back to the same office that we already knew to do the telehealth appointments and to provide urine samples, etc. So, that was really helpful. —a 31-year-old male

Many participants frequently elaborated that embedding PARTNER UP in an SSP provided an opportunity to get healthcare from a provider and staff who they trusted. Participants described that SSP staff are empathetic to the lives of people who use drugs, less judgmental than other healthcare staff, trusted to provide helpful and reliable recommendations to address “their addiction,” and already known to them. Several participants described their experiences with SSP staff—as well as with the PARTNER UP provider—as positive and compared them with their previous interactions with providers and staff at other healthcare settings, which they said had not been positive. Many participants also commented that they were comfortable speaking to the PARTNER UP provider about their medication use because of the provider’s engagement with them, which they described as compassionate, empathetic, and non-judgmental. Participants said:

Not having to go to a regular doctor’s office or anything to get it [is a benefit]. It’s actually easier this way. Because of the stigma of going to the doctor’s office and things like that. You know, a lot of people don’t trust them. A lot of people don’t like going to them. It’s a touchy subject. —a 33-year-old male

So, I really think that it’s important to have a good relationship with your healthcare providers. And I’ve had previous experiences with healthcare providers where I just didn’t feel comfortable, or I wasn’t engaged and actually telling them how I was feeling, or any symptoms that I was experiencing. So, I think that in some ways, it [i.e., PARTNER UP] has been a really good experience just in that sense, because I definitely trust [name of PARTNER UP provider] a lot, and [they] actually seem to care about [their] patients.—a 25-year-old female

One participant explained that he preferred not to go to the SSP for his telemedicine visits because he wanted to avoid “active drug users,” although he planned on continuing his participation in the program.

All participants said they felt comfortable using telemedicine to connect with the PARTNER UP provider. Some said there was no difference in their comfort level between telemedicine and in-person visits, whereas others said they felt more comfortable speaking with a provider using telemedicine than in-person. These participants elaborated that telemedicine visits allowed them to let their guard down and be honest and share more with the provider because they were not in-person and the visits are held in a comfortable location of their choosing. A 45-year-old male described why telemedicine visits feel less intrusive than in-person visits:

[Telemedicine visits are] just better. I like it. Yeah, you don’t feel like it’s interfering. It’s actually like being virtual is easier to be yourself and be truthful…Your guard is up [when in-person].

Some participants also stated that telemedicine visits were less stressful because they do not need to arrange transportation as the visits could be conducted in their homes. A few added that telemedicine eliminates the possibility for stigmatizing behavior from other clinic staff or clients that can occur at in-person clinics. Participants said:

Well, I do like that you can just do it from your bed or wherever you’re at… I think that feels more relaxed as well instead of going into a medical facility where you may get anxious about this or that. —a 30-year-old male

It’s actually a little bit more comfortable because you’re actually in your home, so you’re not jammed up in their little rooms [at a clinic]. —a 35-year-old male

Many participants also noted that the initial in-person meetings with the provider during the first month of PARTNER UP helped to build trust and rapport, which they explained was beneficial when they switched to telemedicine visits for the remaining months of participation.

If I hadn’t met [the provider] in person first, I think I would still feel a little bit less comfortable. But I was lucky enough to meet [them] a couple times…. So, I think that if I hadn’t met [them], I probably would feel a little bit less comfortable.—a 25-year-old female

Participants’ perceptions of the benefits gained from PARTNER UP focused on their improved health and well-being overall. Four participants offered that the program helped them to abstain from using opioids. Three described improved mental health and well-being from participating, and two explained that the program helped them to “get their life back.” A 33-year-old male said:

I've definitely gained more of my life back of being able to have independence from drugs and being able to actually stay off things. I have to make sure that I can get done all the things I need to do.

### Feasibility—perceived ease of participating in PARTNER UP

Most participants reported that they found it feasible to meet weekly with the provider for the first month, with many stating that it helped them become stabilized on MOUD in the program. Several also reported that the subsequent monthly visits were feasible and helpful as they would check in with the PARTNER UP provider and could easily fit the visits into their schedule. Two participants further explained that they were always able to contact the provider whenever needed outside of their scheduled visits if the need arose. Several participants expressed that they had no suggested changes to the weekly and then monthly schedule.

Participants’ ability to keep their scheduled telemedicine visits, however, varied. Half described having no challenges in keeping their scheduled appointment, while half also said they needed to reschedule their appointment at least once during the study. Additionally, due to the telemedicine component, half of participants commented that their ongoing interaction with SSP staff was limited as it primarily focused on maintaining the laboratory assessment schedule. Most, however, expressed their interest in continuing to interact with SSP staff because they were knowledgeable and empathetic.

All participants stressed that telemedicine visits were much easier to attend than in-person visits. Reasons primarily focused on convenience, such that telemedicine visits can be conducted at any location including their homes, and they fit better with their daily schedules. A 33-year-old male explained:

[It’s] also a lot easier [i.e., telemedicine visits]…because I can do it from anywhere. I don’t have to be at a set location.

All participants also said they found it easy to access and use the video conferencing system during the telemedicine visit at the beginning. However, as participants’ time in PARTNER UP progressed, some said they experienced glitches in the video conferencing system that made it difficult or impossible to communicate with the provider using the conferencing platform. In these situations, participants said they communicated with the provider on the telephone (by calling the provider’s work phone or having the provider call them). Additionally, given the option, several participants described not attending their telemedicine visits at the SSP during the first month of participation (after the initial visit) and instead using their own phone to speak with the provider at the location of their choice; half said they had never used the SSP-based computer during any follow-up telemedicine visit. Ultimately, all participants reported preferring the option of using their own device to communicate with the provider versus traveling to the SSP to use a computer for the telemedicine visit, although a few continued using the computer at the SSP for the telemedicine visit because of the opportunity to engage with SSP staff and services. Participants described that using their own device for the telemedicine visits, specifically their telephone, was more convenient, more comfortable, and safer due to potential COVID exposure while traveling around town. Although no participant stated concerns about privacy with embedding PARTNER UP within SSPs, several mentioned that they liked using their own telephone for the telemedicine visit because of the enhanced privacy it provided.

Each participant also described benefits to using the MyChart portal to send messages to the provider. Several noted that the application interface was convenient and easy to use. Participants also expressed their appreciation of having an open “line of communication” with the provider and said MyChart was appropriate to use for questions that did not need an immediate response, as MyChart questions are not immediately viewed by the provider although responses are received within 24 h. A 35-year-old male said:

I normally will do MyChart if it’s not something that I need a response [for] like immediately. [If I need something immediate] I’ll go to the email or I’ll call and leave a voicemail.

Concerns with the feasibility of PARTNER UP were very limited. With respect to ease or difficulty of accessing PrEP and MOUD, a few participants mentioned that accessing Suboxone^®^ at partner pharmacies was challenging and that they would prefer to use a pharmacy closer to them or one that they normally use.

### Intent to use

After the first month of participating, all participants believed they would continue their participation in PARTNER UP until the end of the program. Several said they recognized the benefits of program participation in avoiding the return of drug use, and continued participation would ensure continued access to Suboxone^®^. Some also described participating because they enjoyed meeting with the PARTNER UP provider as well as feeling committed to PARTNER UP or the SSP’s role in the program. Some also mentioned wanting to remain in the program because of their children. A 35-year-old male said:

I’m real confident [that I can stay in the program]. I mean, this is something that I’ve been wanting to do for years. [Interviewer: What is motivating you to do so?] My kids really. I don’t want them to see what I’ve seen growing up. I want them to have a way better life than I ever thought of having growing up.

Toward the end of the program, none of the participants interviewed said they were interested in continuing with PrEP primarily because they did not feel at risk for HIV, although some said they may be interested in taking PrEP in the future if their risk context changed. Nearly all participants said they wanted to continue using Suboxone^®^. A 33-year-old male said:

I definitely want to continue [with Suboxone^®^]…Because I'm on it. I need to stay on it. It's just finding a provider of it that's going to let me just have a month's supply apparently has been the problem right now. And I can't go daily. I have a job at 5:30 in the morning until 3:30.

## Discussion

Participants’ narratives suggest that they found PARTNER UP—a telemedicine-based PrEP and MOUD program embedded within an SSP—both acceptable and feasible. PWID’s established relationships with and trust toward SSP staff provided the foundation for offering a new program that provides access to healthcare providers within the SSP. Participants appreciated that the initial meeting with the PARTNER UP provider was in-person at the SSPs as it allowed for rapport to be established in a trusted space. Our findings also suggest that participants support PrEP and MOUD programs quickly transitioning from an initial in-person visit to telemedicine for follow-up appointments, particularly with the option of using their own device in the location of their choice, as this approach was ultimately preferred. Participants favored using their own telephone as their telemedicine device as it provided the easiest access to the provider. Based on these findings, SSPs are likely effective places to engage with PWID and initiate and integrate healthcare services given PWID’s established relationships with and trust toward SSP staff.

Participants’ narratives emphasized that having direct, easy, and free access to MOUD was the main advantage of PARTNER UP and offering PrEP was less important. Correspondingly, participants indicated their desire to continue their engagement in MOUD programs at the conclusion of PARTNER UP, but they expressed no interest in PrEP unless their risk context changed. Given participants’ history of injecting opioids and interactions with the SSPs, managing their opioid use may be their primary health concern. PWID in other studies have described that “alleviating withdrawal comes first” [9]. Additionally, by accessing unused syringes and other injection equipment at the SSP and reducing their injection frequency due to being on MOUD, participants may believe that they are at lower risk for HIV and therefore may not fully consider HIV risk from sexual transmission and partner risk behavior. While we did not explore sexual behaviors during the IDIs as the focus was on program evaluation, the larger study collected data at multiple time points on sexual behaviors, which suggest that some participants engaged in sexual behaviors that may have placed them at risk [45]. The lack of interest in PrEP may also be due to the small number of PWID who participated in this study; we may see variation in PrEP interest with a larger sample of PWID. Indeed, other studies have shown willingness to take PrEP among larger samples of PWID. For example, 59% of 469 PWID in Los Angeles and San Francisco, California, who were engaged in a non-PrEP prevention intervention reported being willing to take PrEP [6], and 65% of 48 PWID in rural Appalachia who participated in a qualitative study also expressed interest in taking PrEP [7]. Additionally, a larger implementation science pilot is currently underway examining a nurse practitioner-led, same-day MOUD and PrEP program through SSPs [58], which may provide additional insight into interest in PrEP among PWID accessing SSP services. Ultimately, providing information about and access to PrEP and other risk reduction interventions like MOUD at SSPs—whether offered in a combined program or separately—is likely advantageous given the challenges in reaching and providing PWID with health information that could benefit them and others in their social network.

Our findings also suggest that embedding PARTNER UP within an SSP appeared to address several barriers to accessing MOUD and/or PrEP previously expressed by PWID, including stigma, access, and costs [3, 7]. It is worth noting, however, that PARTNER UP had funds available to cover medication costs for participants without insurance and co-pays for participants with insurance. We also provided PrEP- and MOUD-related medical services including provider time and laboratory investigations though the grant. While beneficial to individual participants, financial coverage would be difficult to sustain over time and for a larger number of uninsured participants particularly in states without Medicaid expansion, such as North Carolina. Identifying mechanisms to provide a low threshold program with low costs is critical for long-term success of this type of program—and may become more feasible with funds from the national opioid settlements. Additionally, participants spoke highly of the provider, suggesting that SSPs that wish to embed MOUD and/or PrEP programs partner with healthcare providers who embrace a harm reduction approach.

A strength of our study is that we conducted the IDIs at two time points, which likely reduced recall bias and allowed for a comprehensive understanding of participants’ experiences with PARTNER UP over time. We also chose to implement a program that integrated both PrEP and MOUD in SSPs, and we conducted the pilot program and evaluation in two sites that varied in size and location to determine whether such an approach was acceptable and feasible. However, the number of participants was small in the PARTNER UP program and this evaluation, and a different group of individuals in these two cities or from other cities may have had different experiences and perspectives. Similarly, different participants may have enrolled and we may have learned different perspectives if PARTNER UP focused only on providing PrEP or MOUD individually. Lastly, almost all participants were non-Hispanic White, and it is unknown how PWID of other races and ethnicities may have perceived this program; of note, 77% of SSP participants in North Carolina in 2020–2021 identified as White [38]. Our findings, however, provide useful insights for SSP programs that wish to offer a combined PrEP and MOUD program.

## Conclusions

In conclusion, PARTNER UP participants found the telemedicine-based PrEP and MOUD program acceptable and feasible. Coupled with the outcomes from the larger PARTNER UP evaluation [45, 46], our findings support conducting larger implementation studies to further explore the benefits of embedding both PrEP and MOUD in SSPs.

## Data Availability

Interview transcripts contain potentially identifiable information and therefore are not publicly available due to privacy and ethical restrictions. Codebooks used for data extraction and analysis are available from the corresponding author on reasonable request.

## Abbreviations

IDI: In-depth interview
MOUD: Medications for opioid use disorder
PrEP: Pre-exposure prophylaxis
PWID: People who inject drugs
SSP: Syringe services programs
TDF: Tenofovir disoproxil fumarate
